# EFEMP1通过下调MMP-7表达抑制肺癌细胞生长和侵袭

**DOI:** 10.3779/j.issn.1009-3419.2015.02.08

**Published:** 2015-02-20

**Authors:** 媛媛 郎, 洁 孟, 晓萌 宋, 小军 陈

**Affiliations:** 1 300074 天津，天津儿童医院检验科 Department of Clinical Laboratory, Tianjin Children' s Hospital, Tianjin 300074, China; 2 300070 天津，天津医科大学基础医学院免疫学系 Department of Immunology, Basic Medical College, Tianjin Medical University, Tianjin 300070, China

**Keywords:** EFEMP1, MMP-7, 肺肿瘤, 细胞侵袭, EFEMP1, MMP-7, Lung neoplasms, Invasion

## Abstract

**背景与目的:**

EFEMP1属于fibulin家族成员，是一种与细胞代谢密切相关的重要的细胞外基质蛋白，其在肿瘤的发生发展中的作用尚不清楚。本研究旨在探讨EFEMP1影响肺癌细胞生长和侵袭转移的生物学作用及其机制。

**方法:**

Western blot方法检测肺癌细胞中EFEMP1表达，甲基化特异性PCR（methylation-specific PCR, MSP）方法检测*EFEMP1*在肺癌细胞中启动子区甲基化状态。肺癌细胞中转染EFEMP1后，检测细胞克隆形成及侵袭能力变化，并用Western blot及实时定量PCR检测MMP-7表达，Luciferase实验检测EFEMP1对基质金属蛋白酶7（matrix metalloproteinase-7, MMP-7）报告质粒的影响。

**结果:**

Western blot结果显示肺癌细胞中EFEMP1表达下降，MSP分析结果说明A549和H1299中*EFEMP1*启动子区存在甲基化位点，5-aza-2’-deoxycytidine处理后，EFEMP1表达升高。A549和H1299转染EFEMP1后细胞克隆形成能力以及侵袭活性明显下降，MMP-7蛋白表达下调。Luciferase实验结果显示EFEMP1可以抑制MMP-7报告质粒的表达活性。

**结论:**

EFEMP1是一种肺癌生长和侵袭的抑制因子，由于表观遗传学的改变，其在肺癌细胞中表达下降，通过上调MMP-7的表达促进肺癌细胞的侵袭转移。

## Abstract (en)

**Background and objective:**

EFEMP1, a member of fibulin family proteins, is a very important extracellular matrix protein which is involved in cell metabolism and its role in tumor occurrence and progression is still poorly understood. The aim of this study is to investigate the functional effect and mechanism of EFEMP1 in lung cancer cell growth and invasion.

**Methods:**

EFEMP1 expression in lung cancer cells was determined by Western blot. The promoter methylation status of EFEMP1 was detected by methylation-specific PCR (MSP). After transfection of control or EFEMP1 vector in lung cancer cells, the ability of colony formation and invasion was detected by colony formation experiment and matrigel invasion method. Western blot and real-time PCR were used to detect matrix metalloproteinase-7 (MMP-7) expression. Luciferase assay was used to detect expression of MMP-7 reporter construct transfected with or without EFEMP1 in lung cancer cells.

**Results:**

Western blot result showed EFEMP1 expression was downregulated in lung cancer cells. The promoter region of *EFEMP1* was methylated in A549 and H1299 and after treatment with 5-aza-2'-deoxycytidine, the EFEMP1 expression was upregulated. The growth and invasion of A549 and H1299 were all significantly suppressed by transfecting with EFEMP1 and the MMP-7 expression was dowanregulated by EFEMP1 as well. Expression activity of MMP-7 reporter construct was decreased by cotransfecting with EFEMP1.

**Conclusion:**

Collectively, these results suggest that EFEMP1 functions as a suppressor of lung cancer growth and invasion. Epigenetic silencing of EFEMP1 promotes lung cancer invasion and metastasis by activating MMP-7 expression.

在众多癌症中，肺癌的死亡率位居第一位，通常5年生存率低于20%，大多数患者死于肺癌转移^[1]^。但是，迄今为止，肺癌的侵袭与转移机制尚未阐明。Fibulin家族蛋白是一种表达广泛的细胞外基质蛋白（extracellular matrix, ECM），迄今为止共发现七个成员（fibulin 1-7），它们在结构上具有相似性，都有数个重复的表皮生长因子样结构域和一个独特的C末端结构域，并且在调节细胞与细胞间，细胞与外基质间相互联系中起重要作用^[2]^。越来越多的证据^[3]^表明fibulin家族蛋白在癌症侵袭转移过程中也起重要作用，它们在多种癌症细胞中表达异常，可以促进或抑制癌细胞侵袭转移。

包含表皮生长因子的fibulin类细胞外基质蛋白1（EGF containing fibulin-like extracellular matrix protein 1, EFEMP1），即fibulin 3，是一种与细胞代谢密切相关的重要细胞间粘附分子^[2, 3]^。本研究旨在探讨EFEMP1影响肺癌细胞生长和侵袭转移的生物学作用及其机制，以期为阐明肺癌侵袭转移机制及为肺癌的靶向治疗提供重要线索。

## 1 材料与方法

### 1.1 主要试剂和仪器

本研究所用肺癌细胞系购于ATCC，RPMI-1640培养液、胎牛血清和胰蛋白酶均购自Gibco公司，EFEMP1抗体购自Abcam，基质金属蛋白酶7（matrix metalloproteinase-7, MMP-7）抗体购自EMD BioSciences，实时定量PCR试剂盒购于RT-PCR试剂盒购自Promega公司，基因组DNA提取试剂盒购自Qiagen公司，MSP试剂盒购于ZYMO Research，5-aza-2’-deoxycytidine购于Sigma，Luciferase检测试剂购于Promega公司，ECL免疫印迹底物试剂盒购自Millipore公司，Transwell培养板购于BD Biosciences，PCR仪：Thermo荧光检测仪：Perkinelmer。

### 1.2 细胞培养

本研究所用肺癌细胞系培养于含10%胎牛血清的RPMI-1640培养基，外加100 U/mL青霉素和100 μg/mL链霉素，置于37 ℃、5%CO_2_培养箱中培养。去甲基化实验中，细胞培养于5 μmol/L 5-aza-2’-deoxycytidine，共培养6天，其中第1、2、3天换新鲜培养液。

### 1.3 基因组DNA提取和MSP

肺癌细胞系基因组DNA用Qiagen公司的QIAamp DNA Blood minikit试剂盒提取。取0.25 μg基因组DNA外加1.0 μg鲑鱼精子DNA用ZYMO Rearch公司的EZ DNA Methylation Gold kit进行重亚硫酸盐处理，修饰后的基因组DNA溶于40 μL蒸馏水。用修饰后的基因组DNA做甲基化特异性PCR（methylation-specific PCR, MSP），其中甲基化*EFEMP1*启动子用以下引物扩增：5’-GTAGTTTTAGGGGATCGTCGC-3’/5’-TCCCCGACACGCTACCTTCG-3’。非甲基化*EFEMP1*启动子用以下引物扩增：5’-GAGTAGTTTTAGGGGATTGTTGT-3’/5’-TCCCCAACACACTACCTTCA-3’。

### 1.4 Real time-PCR

利用Promega总RNA提取试剂盒提取12孔板中细胞总RNA，检测总质量，用Invitrogen公司逆转录酶将10 μg总RNA逆转录为第一链cDNA，用下列引物进行MMP-7的touchdown实时定量PCR扩增：5’-AAACTCCCGCGTCATAGAAAT-3’/5’CCCTAGACTGCTACCATCCG-3’。

### 1.5 质粒和siRNA转染

根据Lipofectamine 2000说明书进行质粒转染和siRNA转染，构建稳定表达EFEMP1细胞株时，先转染EFEMP1表达质粒，后用0.4 mg/mL G418对A549和H1299进行筛选，并用细胞裂解液进行Western blot鉴定。MMP-7 siRNA使用Dharmacon公司合成的MMP-7 877（GCACUGUUCCUCCACUCCA）。

### 1.6 克隆形成实验

细胞在12孔板转染EFEMP1或对照质粒48 h后转入35 mm培养皿中继续培养，同时培养基中加入终浓度0.4 mg/mL G418进行筛选，11 d-14 d后对培养皿中细胞进行结晶紫染色，并计数着色细胞。

### 1.7 基质胶侵袭实验

肺癌细胞培养于BD公司的6孔trans-well培养板，上层培养室的8 μm滤膜覆盖以1:6稀释的基质胶，每个培养孔均匀铺以约2×10^6^个细胞，培养36 h后，穿越滤膜到达培养孔底部的细胞用福尔马林固定，并用结晶紫染色。每孔随机计数10个视野（×200）的细胞数。

### 1.8 荧光素酶检测

已构建的MMP-7荧光素酶报告质粒（pBV-Luc载体为母板，通过插入264 bp的MMP-7启动子区片段构建而成），其与pcDNA或EFEMP1，以及β-gal共转染肺癌细胞，24 h后收集细胞，按Promega公司LAR试剂盒说明进行处理，并检测荧光素酶相对表达活性。

### 1.9 统计学方法

使用GraphPad Prism Ⅳ软件进行分析统计，计量资料采用*t*检验比较组间差异，以*P* < 0.05为差异有统计学意义。

## 2 结果

### 2.1 EFEMP1表达及启动子区甲基化分析

Western blot检测几种肺癌细胞系的EFEMP1表达，A549和201T中表达较低，1288.88T中表达较高，H1299中几乎无表达，而稳定表达EFEMP1的H1299细胞则可检测到明显表达（图 1A）。用MSP检测A549和H1299全基因组中*EFEMP1*启动子区的甲基化状态，由图 1B可见在A549和H1299细胞中甲基化组均可观察到条带，说明在这两种细胞系中EFEMP1启动子区均有甲基化存在。进而用5-aza-2’-deoxycytidine处理细胞，RT-PCR结果显示处理后EFEMP1表达均升高（图 1C）。

**1:**
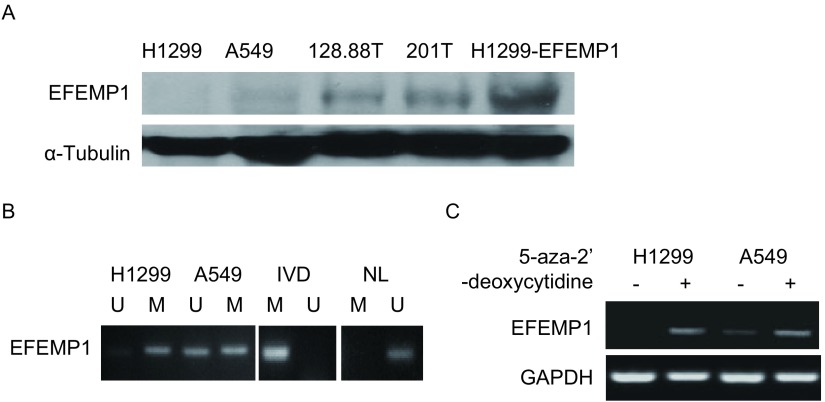
H1299和A549中由于*EFEMP1*启动子区甲基化导致表达下降。A：不同肺癌细胞的EFEMP1表达；B：EFEMP1在A549和H1299中甲基化状态分析；C：RT-PCR分析5-aza-2’- deoxycytidine处理前后A549和H1299中EFEMP1的表达。 Silence of *EFEMP1* in lung cancer by promoter hypermethylation. A: EFEMP1 expression in four lung cancer cell lines and EFEMP1-overexpressing H1299; B: *EFEMP1* methylation status in A549 and H1299 was analy zed by MSP. NL and IVD were negative and positive controls; C: EFEMP1 expression in A549 and H1299 with or without 5-aza-2'-deoxycytidine treatment was determined by RT-PCR. M: methylation; U: unmethylation; MSP: methylation-specific PCR.

### 2.2 克隆形成以及Transwell侵袭实验

A549和H1299分别转染EFEMP1和对照质粒，48 h后消化转入培养皿中，并用G418开始筛选，11 d-14 d后结晶紫染色，发现转染EFEMP1细胞组克隆形成数明显少于对照组（图 2A）。除了克隆形成外，用Transwell侵袭实验发现转染EFEMP1细胞组侵袭性也明显小于对照组（图 2B）。

**2:**
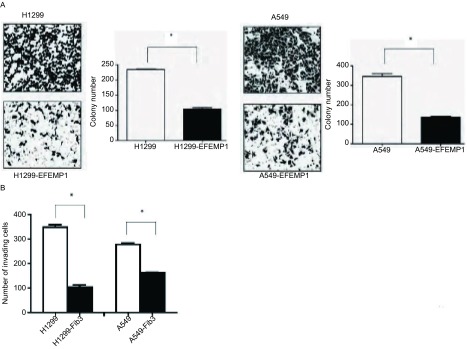
EFEMP1的表达抑制肺癌细胞的侵袭能力。A：EFEMP1表达抑制A549和H1299的克隆形成能力；B：基质胶侵袭实验分析转染EFEMP1后A549和H1299细胞的侵袭能力。^*^：*P* < 0.01 Student’s *t*-test。 EFEMP1 expression suppressed lung cancer cell invasion. A: Colony formation of A549 and H1299 cells transfected with EFEMP1 or the control pcDNA vector; B: Matrigel invasion assay was used to analyze the invasion of H1299 and A549 cells with or without EFEMP1 expression. ^*^: *P* < 0.01 Student's *t*-test.

### 2.3 EFEMP1通过抑制MMP-7表达而降低肺癌细胞侵袭性

A549和H1299转染EFEMP1，Western blot检测12 h、24 h的MMP-7表达量，发现比对照组明显降低，并用实时定量PCR检测MMP-7的相对表达量（图 3A）。EFEMP1和对照质粒分别与MMP-7荧光素酶报告质粒共转染A549和H1299细胞，24 h后检测各组的相对荧光素酶活性，我们发现EFEMP1会明显抑制报告质粒的荧光素酶活性（图 3B）。

**3:**
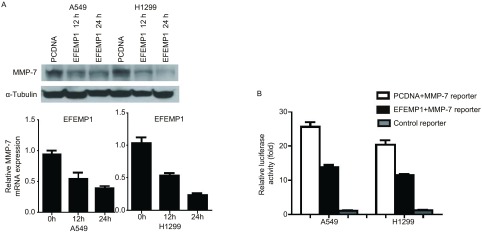
EFEMP1通过下调MMP-7的表达抑制肺癌细胞侵袭。A：EFEMP1转染肺癌细胞后，Western blot分析12 h、24 h MMP-7的表达，实时定量PCR分析MMP-7表达量变化；B：荧光素酶活性实验分析EFEMP1表达下调MMP-7报告质粒的荧光素酶表达。 EFEMP1 suppressed lung cancer cell invasion by downregulating MMP-7 expression. A: Western blot analysis of MMP-7 expression in A549 and H1299 cells at 12 h and 24 h after transfection with EFEMP1 or the control empty vector. MMP-7 expression was confirmed by real-time PCR; B: Luciferase activities of MMP-7 reporter construct were determined 24 h after transfection with EFEMP1. MMP-7: matrix metalloproteinase-7.

### 2.4 MMP-7表达沉默降低肺癌细胞的侵袭性

用MMP-7 siRNA转染肺癌细胞A549和H1299，36 h后收集细胞，Western blot检测MMP-7的表达明显下降（图 4A），同时基质胶侵袭实验检测MMP-7沉默组细胞其侵袭性明显低于对照组（图 4B）。其细胞克隆形成数也明显少于对照组（图 4C）。

**4:**
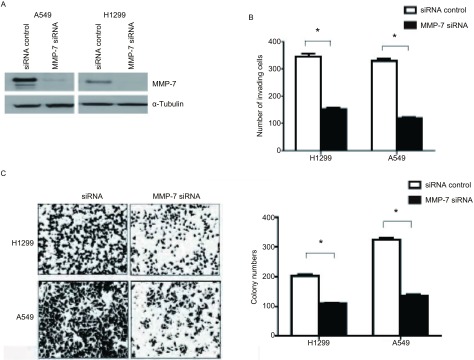
MMP-7表达沉默后降低肺癌细胞的侵袭性。A：siRNA沉默后，Western blot检测MMP-7表达；B：siRNA沉默后，基质胶侵袭实验分析A549和H1299的侵袭性；C：MMP-7经siRNA沉默后，克隆形成实验检测A549和H1299的增殖能力。^*^：*P* < 0.01 Student’s *t*-test。 Silence of MMP-7 expression decreased the invasiveness in lung cancer cells. A: MMP-7 expression was analyzed by Western blot 36 h after siRNA transfection in A549 and H1299; B: Cell invasion was analyzed by Matrigel assay 36 h after siRNA transfection; C: Colony formation of A549 and H1299 cells transfected with MMP-7 siRNA or the siRNA control. ^*^: *P* < 0.01 Student's *t*-test.

## 3 讨论

Fibulin家族包括七个成员^[3]^（fibulin 1-7），是一组细胞外基质蛋白，它们的结构高度同源，具有相似的结构域组成，包括结构域Ⅰ、Ⅱ和Ⅲ。Fibulin家族表达广泛，定位于细胞基底膜、基质和ECM等，介导细胞与细胞、细胞与基质之间的相互联系，在器官和血管形成过程中使ECM正确组织与稳定。它们与内皮细胞相互作用，参与组织器官重塑和修复等机体正常功能。

Fibulin蛋白在肿瘤组织中异常表达，与癌症的发生发展密切相关^[3]^。大量研究表明EFEMP1蛋白在结肠癌^[4]^、肝癌^[5]^、前列腺癌^[6]^、鼻咽癌^[7]^等癌症细胞中表达下调或缺失，导致肿瘤细胞侵袭转移能力提高，然而EFEMP1在肿瘤进展中的作用并不完全相同，它的作用依据细胞种类和微环境的不同而表现出不同方式^[3, 8]^。比如在宫颈癌^[9]^、脑癌^[10]^和胰腺癌^[11]^中，EFEMP1表达明显上升，对肿瘤的发生发展起到促进的作用。

我们之前和现在的研究结果证实EFEMP1在肺癌中常表达下调（图 1）^[12]^，EFEMP1在大多数正常肺组织（80%）中表达，但在约40%的肺癌组织中表达缺失，造成这种现象的部分原因就是EFEMP1在肺癌细胞中其启动子区甲基化，抑制其正常表达。

*EFEMP1*在肺癌中的启动子区甲基化状态可以作为诊断肺癌的一种生物学指标^[13]^。最新的研究^[14]^证明血清中的EFEMP1水平可以作为恶性间皮细胞瘤诊断的重要指标，因为它较正常人水平明显升高。

本研究发现EFEMP1在肺癌细胞中的表达下降对于肺癌侵袭转移起到重要的作用，EFEMP1在肺癌细胞中可起到抑制生长和侵袭的作用，在EFEMP1表达缺失的肺癌细胞中转染EFEMP1可以明显降低肺癌细胞的克隆形成能力和侵袭能力（图 3）。EFEMP1在肺癌中抑制癌细胞生长和侵袭的作用是通过抑制MMP-7的表达来介导，MMP-7在肺癌细胞的侵袭过程中起重要作用^[15, 16]^，其在肺癌中往往高表达，并提示不良预后^[17]^。EFEMP1在肺癌细胞中表达下降，转染EFEMP1后，MMP-7表达下调（图 4），并且EFEMP1在肺癌细胞中存在启动子区甲基化现象。

Fibulin家族中，与EFEMP1结构相似的fibulin-5在肺癌细胞中也可以下调MMP-7的表达^[18]^，另外除了MMP-7，EFEMP1、fibulin 5在其生物学功能上还可作用于其他的MMP/TIMP家族蛋白，例如EFEMP1是timp-3的结合配体^[19]^，fibulin-5在某些肿瘤中可以调节MMP-2、MMP-3、timp-1、timp-3等的表达^[20]^，这些研究结果说明不同的fibulin家族成员在肿瘤细胞中都可协同或独自调节MMP/TIMP家族蛋白，从而影响肿瘤细胞的侵袭能力。本文中，我们并没有发现在肺癌细胞中EFEMP1可以调控除MMP-7之外的MMP/TIMP家族蛋白（结果未显示）。

我们的实验数据证明了EFEMP1蛋白在肺癌侵袭转移过程中起到抑制作用，并可下调MMP-7表达，而MMP-7表达以及MAPK信号通路激活等都是上皮间质细胞转化（epithelial mesenchymal transition, EMT）过程的重要机制，肿瘤相关的MMP蛋白本身可以激活肿瘤EMT过程^[21, 22]^，故我们相信EFEMP1在肺癌中的表达下调，并且上调MMP-7表达，这些都是肺癌细胞EMT过程的重要事件。

总之，我们的实验结果揭示了EFEMP1在肺癌中通过抑制MMP-7表达从而抑制癌细胞侵袭的生物学功能，下一步，我们将深入研究该过程的具体分子机制及信号通路，研究结果将有助于为肺癌转移的靶向治疗提供新的作用靶点。
